# Healthy and Resilient Cereals and Pseudo-Cereals for Marginal Agriculture: Molecular Advances for Improving Nutrient Bioavailability

**DOI:** 10.3389/fgene.2020.00049

**Published:** 2020-02-27

**Authors:** Juan Pablo Rodríguez, Hifzur Rahman, Sumitha Thushar, Rakesh K. Singh

**Affiliations:** Crop Diversification and Genetics Program, International Center for Biosaline Agriculture, Dubai, United Arab Emirates

**Keywords:** orphan crops, nutrient-rich crops, pseudo-cereals, molecular profiles, food and nutritional security, healthy crops, marginal environment

## Abstract

With the ever-increasing world population, an extra 1.5 billion mouths need to be fed by 2050 with continuously dwindling arable land. Hence, it is imperative that extra food come from the marginal lands that are expected to be unsuitable for growing major staple crops under the adverse climate change scenario. Crop diversity provides right alternatives for marginal environments to improve food, feed, and nutritional security. Well-adapted and climate-resilient crops will be the best fit for such a scenario to produce seed and biomass. The minor millets are known for their high nutritional profile and better resilience for several abiotic stresses that make them the suitable crops for arid and salt-affected soils and poor-quality waters. Finger millet (*Eleucine coracana*) and foxtail millet (*Setaria italica*), also considered as orphan crops, are highly tolerant grass crop species that grow well in marginal and degraded lands of Africa and Asia with better nutritional profile. Another category of grains, called pseudo-cereals, is considered as rich foods because of their protein quality and content, high mineral content, and healthy and balance food quality. Quinoa (*Chenopodium quinoa*), amaranth (*Amaranthus* sp.), and buckwheat (*Fagopyrum esculentum*) fall under this category. Nevertheless, both minor millets and pseudo-cereals are morphologically different, although similar for micronutrient bioavailability, and their grains are gluten-free. The cultivation of these millets can make dry lands productive and ensure future food as well as nutritional security. Although the natural nutrient profile of these crop plant species is remarkably good, little development has occurred in advances in molecular genetics and breeding efforts to improve the bioavailability of nutrients. Recent advances in NGS have enabled the genome and transcriptome sequencing of these millets and pseudo-cereals for the faster development of molecular markers and application in molecular breeding. Genomic information on finger millet (1,196 Mb with 85,243 genes); *S. italica*, a model small millet (well-annotated draft genome of 420 Mb with 38,801 protein-coding genes); amaranth (466 Mb genome and 23,059 protein-coding genes); buckwheat (genome size of 1.12 Gb with 35,816 annotated genes); and quinoa (genome size of 1.5 Gb containing 54,438 protein-coding genes) could pave the way for the genetic improvement of these grains. These genomic resources are an important first step toward genetic improvement of these crops. This review highlights the current advances and available resources on genomics to improve nutrient bioavailability in these five suitable crops for the sustained healthy livelihood.

## Introduction

All foods have uniqueness in their composition, with a specific range of macro- and micronutrients in different combinations. Some foods are rich in carbohydrate (rice, wheat, maize, etc.), protein (pulses, spinach), fat (oilseeds, groundnut), or mineral (pearl millet, etc.), while some are nutrient-dense and have optimum combinations of nutrients with good digestibility (most of the minor millets, quinoa, etc.). These nutrient-dense foods with a proper mix of nutrients and high bioavailability are sometimes designated as superfoods, but the irony is that most of these are also considered as orphan crops because of lower cropped area, low demand, and low consumption. Minor millets and pseudo-cereals come under the category of underused and neglected crops.

Every day, arable non-stress areas are being converted into marginal lands at an alarming rate, which may further increase under the climate change scenario if business as usual (RCP8.5) continues without arresting the trend of global warming as per the IPCC AR5 (Porter et al., 2014). Recent studies based on compiled data from 53 countries with serious, alarming, or seriously alarming conditions clearly show that more than 50% (27 countries) had decreased consumed calories due to mean climate change (Ray et al., 2019). Although the productivity of major crops has been predicted to be lower under marginal environments, the good news is that the nutrient-rich underused neglected crops are very resilient to harsh environments (drought, salinity, and extreme temperature) and yield well with limited resources (Mabhaudhi et al., 2019). As per FAO estimates (FAO, 2017), most of the food needed for 2 billion more mouths by 2050 has to come from marginal environments. A “Next-Generation Green Revolution” is required to achieve food security, which is a much broader and systems-based approach, and this has to come from the areas that were left out of the first Green Revolution, to achieve future food security in a more sustainable way across all spectrums of society (Nusslein et al., 2016; Dhankher and Foyer, 2018). Huge scope exists for the genetic improvement of these crops but, unfortunately, only limited research programs are undertaking focused research on such crops worldwide. Small and complex flower shape has compounded the difficulty to handle these crops; hence, few genetic studies are being undertaken. This is one of the major factors for slowdown of the research on developing molecular markers for the agronomically important traits to be used in breeding programs. Most genomics studies focus on population structures, grouping, and evolution. The current paper is a review of the scientific work on neglected but nutrient-rich crops such as quinoa, amaranth, finger millet, foxtail millet, and buckwheat. In recent years, progress in addressing all forms of malnutrition has seen a declining trend, alarmingly slow, with >150 million children still stunted (Global Nutrition Report, 2018), but the movement has increased awareness among people and hence increased health-conscious people demanding healthy food relatively more often. This review will deal with five underused but nutritionally important and rich crops: finger millet (*Eleucine coracana*), foxtail millet (*Setaria italica*), quinoa (*Chenopodium quinoa*), amaranth (*Amaranthus* sp.), and buckwheat (*Fagopyrum esculentum*).

## Bioavailability and Healthy Food

Bioavailability, in simple terms, refers to whatever is absorbed out of ingested food and goes into the bloodstream. Many factors could affect this process, but the prominent ones are the original profile of food, processing of food, and digestion efficiency. Nutritional composition is the mix of macronutrients (carbohydrate, fat, and protein) and micronutrients (minerals and vitamins) within a product, but their absorption efficiency in the body depends on the ability of food to be digested easily. Food that is absorbed easily maintains or improves health and energy status by providing appropriate macro- and micronutrients in balanced form and is thus called healthy food. Neglected or underused crops such as minor millets and pseudo-cereals were part of the common diet of ancient cultures but slowly, after the Green Revolution, the higher availability and accessibility of rice, wheat, and maize overtook these neglected crops and started providing >60% of the calorific intake through these three crops only, thus starting to create a nutrient-imbalanced diet. Not many studies exist on the bioavailability of the nutrients provided by nutrient-dense underused crops.

## Bioavailability and Improvement in Molecular Genetics

### Finger Millet

Millets serve as a good food source of carbohydrates, proteins, minerals, and vitamins. Although they are the basic food ingredient in the diets of millions of people living in the semi-arid and arid regions of the world, they are still sometimes referred to as orphan crops or even lost crops. These neglected crops are mostly cultivated in developing countries and their world production statistics show low volumes vis-à-vis other popular food crops. These neglected crops are important because of their contribution to biodiversity and climatic resilience, their rich nutrition profile, and their means of livelihood of the poor in various parts of the world (Belton and Taylor, 2004). Finger millet is one of the most efficient crops for nitrogen use efficiency (NUE) and it can grow well with less water requirement, hence well suited to semi-arid climates (Gupta et al., 2017). It is also responsive to nutrients but has the ability to do well under limited resources (Gull et al., 2014). The most important part is its excellent storing capacity without deterioration even with significant insect and pest attacks. This has earned it the popular name of “famine crop” as it can resist storage pests for as long as 10 years, ensuring a year-round food supply (Mgonja et al., 2007).

Among the millet crops, six crops are called minor millets due to their small size: finger millet (*Eleusine coracana* (L.) Gaertn.), foxtail millet (*S. italica* (L.) P. Beauv.), kodo millet (*Paspalum scrobiculatum* L.), proso millet (*Panicum miliaceum* L.), barnyard millet (*Echinochloa* spp.), and little millet (*Panicum sumatrense* Roth). All these small minor millets are known for their unique nutritional composition and resilience (Kumar et al., 2018). Only foxtail and finger millet from this group will be discussed here. Finger millet is relatively popular in India and many countries in Africa because of its resilience and nutrient-dense grain profile. It has been promoted in Africa to reduce anemic incidence in children (Tripathi and Patel, 2010; Udeh et al., 2017). The nutraceutical importance of finger millet lies in its high content of calcium (0.38%), protein (6–13%), dietary fiber (10–18%), carbohydrate (65–75%), and minerals (2.5–3.5%). Another quality of finger millet is that it is gluten free with low glycemic index (GI) hence suitable for the people suffering from gluten intolerance/coeliac disease as well as diabetes (**Tables 1** and **2**). It is rich in ergocalciferol (vitamin D) and essential amino acids (EAA) such as valine, phenyl-alanine, leucine, and histidine (**Tables 3** and **4**). Besides these important nutrients, it has phytates (0.48%), tannins (0.61%), phenolic compounds (0.3–3.0%), and trypsin inhibitory factors that affect the bioavailability of nutrients; that is why proper processing is important to exploit its positive nutritional qualities (Devi et al., 2014) (**Tables 5** and **6**). Chauhan and Sarita (2018) have reported increased bioavailability of minerals such as Fe and P and vitamins upon grain processing before consumption. Phytates and tannins have negative effects on the bioavailability of nutrients, but processing at germination and little fermentation of grains increase the availability of minerals, amino acids, and free sugars, along with digestibility (Sripriya et al., 1997). Finger millet is mostly consumed as flour, but its processing through germinating and fermenting makes its iron content much higher in grains (Tatala et al., 2007). Seed germination of finger millet can increase the bioavailability of iron from 0.75 to 1.25 mg/100 g and is a potential alternative to mitigate anemia (Tatala et al., 2007). The pre-processing of grains can drastically reduce the impact of anti-nutrients and can improve iron bioavailability and bioactive compounds, which are confirmed by several scientific studies (Tatala et al., 2007; Hithamani and Srinivasan, 2014; Udeh et al., 2017) (**Tables 5** and **6**).

**Table 1:** Proximate component profile of grains.

	Crude protein	Total fat	Dietary fiber			Carbohydrate	Gluten presence	GI
			Total	Insoluble	Soluble		
Finger millet (*Eleucine coracana*)	7.16	1.92	11.18	9.51	1.67	66.82	No	Low
Foxtail millet (*Setaria italica*)	8.92	2.55	6.39	4.29	2.11	66.19	No	Low
Quinoa (*Chenopodium quinoa*)	13.11	5.50	14.66	10.21	4.46	53.65	No	Low
Amaranth (*Amaranthus* spp.)	14.59	5.74	7.02	5.76	1.26	59.98	No	High
Buckwheat (*Fagopyrum esculentum)*	13.25	3.40	10.00	–	–	71.50	No	Low

All values are expressed in percentage of edible portion. Hyphens (-) in the tables represent either below detectable limit or not reported. Compiled from Johnson and Croissant (1985); Gopalan et al. (1989); Longvah (2017); Dayakar et al. (2017); USDA National Nutrient Database for Standard Reference. https://fdc.nal.usda.gov/(accessed on October 28, 2019).

GI, glycemic index; (low <55; intermediate 55–70; and high >70).

**Table 2:** Mineral composition in grains (mg/100 g).

		Finger millet	Foxtail millet	Quinoa	Amaranth	Buckwheat
Calcium	Ca	364	15.27	198	181	18
Copper	Cu	0.67	0.26	0.48	0.81	1.10
Iron	Fe	4.62	2.34	7.51	9.33	2.20
Magnesium	Mg	146	122	119	325	231
Manganese	Mn	3.19	0.33	1.77	5.29	1.30
Phosphorus	P	210	101	212	374	347
Potassium	K	443	94	474	433	460
Sodium	Na	4.75	3.35	4.50	2.70	1.00
Zinc	Zn	2.53	1.65	3.31	2.66	2.40

Compiled from Johnson and Croissant (1985); Gopalan et al. (1989); Longvah (2017); Dayakar et al. (2017); USDA National Nutrient Database for Standard Reference. https://fdc.nal.usda.gov/(accessed on October 28, 2019).

**Table 3:** Amino acid profile (g/100 g protein).

		Finger millet	Foxtail millet	Quinoa	Amaranth	Buckwheat
Alanine	ALA	6.71	11.00	4.35	4.26	4.50
Arginine	ARG	4.33	3.18	7.85	7.77	9.70
Aspartic acid	ASP	6.40	5.61	8.40	12.57	11.30
Glutamic acid	GLU	20.22	18.25	13.75	16.12	18.60
Glycine	GLY	3.59	3.12	4.80	8.50	6.30
Proline	PRO	5.42	7.33	5.67	3.76	3.80
Serine	SER	4.81	5.50	4.56	7.79	4.70
Tyrosine	TYR	3.37	3.87	1.98	2.85	2.10
Histidine	HIS	2.37	2.14	2.98	1.86	2.70
Isoleucine	ILE	3.70	4.55	3.75	2.82	3.80
Leucine	LEU	8.86	11.96	6.08	4.83	6.40
Lysine	LYS	2.83	1.42	5.55	5.45	6.10
Methionine	MET	2.74	2.69	2.24	1.86	2.50
Cystine	CYS	1.48	1.92	1.85	1.60	1.60
Phenyl-alanine	PHE	5.70	6.27	4.35	3.98	4.80
Threonine	THR	3.84	3.89	3.01	3.02	3.90
Tryptophan	TRP	0.91	1.32	1.25	1.05	2.00
Valine	VAL	5.65	5.49	4.55	4.34	4.70

Compiled from Pomeranz and Robbins (1972); Johnson and Croissant (1985); Gopalan et al. (1989); Ikeda and Kishida (1993); Longvah (2017); Dayakar et al. (2017); USDA National Nutrient Database for Standard Reference. https://fdc.nal.usda.gov/(accessed on October 28, 2019).

**Table 4:** Vitamins in minor millets and pseudo-cereals.

		Unit	Finger millet	Foxtail millet	Quinoa	Amaranth	Buckwheat
Fat soluble	α-Ergocalciferol (vit. D)	µg	41.46	–	–	0.04	–
	α-Tocopherol (vit. E)	mg	0.16	–	2.08	1.92	0.32
	Phylloquinones (vit. K1)	µg	3.00	–	2.00	–	7.00
Water soluble	Thiamine (vit. B_1_)	mg	0.37	0.59	0.83	0.04	0.42
	Riboflavin (vit. B_2_)	mg	0.17	0.11	0.22	0.04	0.19
	Niacin (vit. B_3_)	mg	1.34	3.20	1.70	0.45	6.15
	Pantothenic acid (vit. B_5_)	mg	0.29	0.82	0.62	0.24	0.44
	Vit. B_6_	mg	0.05	–	0.21	0.50	0.58
	Biotin (vit. B_7_)	µg	0.88	–	0.62	1.92	–
	Folates (vit. B_9_)	µg	34.66	–	1.73	27.44	54.00

All values are expressed per 100 g edible portion; all blank spaces (-) in the tables represent either below detectable limit or not reported. Compiled from Johnson and Croissant (1985); Gopalan et al. (1989); Longvah (2017); Dayakar et al. (2017); USDA National Nutrient Database for Standard Reference. https://fdc.nal.usda.gov/(accessed on October 28, 2019).

**Table 5:** Effects, mechanism, and process of increasing bioavailability of cereals and pseudo-cereal grains.

Crop	Effects	Mechanism	Process to increase bioavailability	References
Finger millet	Reduction in viscosity of weaning food	NA	Malting	Seenappa (1988)
	Eliminate stickiness of cooked millet	NA	Parboiling	Desikachar (1975)
	Flour quality can be increased	NA	Decortication	Geervani and Eggum (1989)
	Loss of protein, mineral, and fiber content	NA	Dehulling, soaking, and cooking	Panwal and Pawar (1989)
	Increase in *in vitro* protein digestibility (IVPD)	NA	Dehulling of seeds	Ramachandra et al. (1977)
	Effective removal of polyphenols and phytates	NA	Dehulling followed by soaking	Pawar and Parlikar (1990)
	Improve recovery of soluble protein and its digestibility *in vitro*	NA		
Foxtail millet	Significant increase in extractability of calcium, phosphorus, iron, zinc, and copper	NA	Roasting	Gahlawat and Sehgal (1995)
	Digestibility and biological values increased	NA	Fortified with lysine	Ganapathy et al. (1957)
	Highest concentration of thiamine, vitamin E, and stearic and linoleic acid	NA	NA	Bandyopadhyay et al. (2017)
	Loss of protein, mineral, and fiber content	NA	Dehulling/soaking/cooking	Pawar and Machewad (2006)
	Increase in percentage of ionizable iron and soluble zinc	By the removal of polyphenols and breaking down of polyphenols-protein-minerals		
	Two types of fatty acid patterns observed	Glutinous and non-glutinous varieties	NA	Taira (1984)
	High amount of protein (11%) and fat (4%). The protein fractions are represented by albumins and globulins (13%), prolamins (39.4%), and glutelins (9.9%). It is thus recommended as an ideal food for diabetics.	NA	NA	Saleh et al. (2013)
Quinoa	Higher lysine and methionine content	NA	NA	Bhargava et al. (2003)
	Increased protein efficiency ratio (PER)	NA	Cooking	Mahoney et al. (1975)
	Increased *in vitro* digestibility	NA	Cooking, autoclaving, drum drying	Ruales and Nair (1993a)
	Changes in total dietary fiber content	NA	Thermal treatment	
	Decreased oil absorption capacity of quinoa flour	NA	Adding salt	Ogungbenle (2003)
	Rich source of antioxidants	NA	NA	Debski et al. (2013)
	Considered as golden grain because of its nutritional properties. Thus, NASA integrated this into the food of astronauts.	NA	NA	Rojas et al. (2010)
	Helps to reduce fatty acid uptake and esterification in adipocyte	NA	NA	Foucault et al. (2012)
	Significant impact on the chemical profile of quinoa flour	NA	Extrusion and roasting	Brady et al. (2007)
	Helps to degrade phytate in flour	Degradation of phytate in pseudo-cereal flours may depend on the activation of endogenous phytase and on the production of exogenous phytase by starter culture	Fermentation	Castro-Alba et al. (2019)
	Improved mineral availability of flours	Fermentation with *Lactobacillus plantarum*	Fermentation	
	Higher level of phytate degradation in quinoa grains	NA	Abrasion process to eliminate saponins	
	Rich source of phytoecdysteroids	NA	NA	Kumpun et al. (2011)
	Anabolic, performance enhancing, anti-osteoporotic, wound-healing properties	Phytoecdysteroids	NA	Graf et al. (2014)
	Reduction in phytate content	NA	Germination, cooking, and fermentation	Valencia et al. (1999)
	Increased iron solubility	NA	Soaking and germination	
Amaranth	Reduces bioavailability of calcium and magnesium	Oxalates	Cooking/popping	Arêas et al. (2016)
	Presence of antinutritional factors	
	Reduces bioavailability of carbohydrates	Inhibition of amylases contributing to the reduction of glucose levels in blood
	Reduction in blood cholesterol level	Decrease the solubility of cholesterol micelles by Amaranth oil
	High-protein amaranth flour (HPAF)	enzymatic hydrolysis	Liquefaction/saccharification	Guzmán-Maldonado and Paredes-López (1998)
	Improves grain nutrient profile	NA	Malting/germination	Hejazi et al. (2016)
	Increases availability of proteins as well as free amino acid components	NA	Sprouting	Paredes-Lopez and Mora-Escobedo (1989)
	Reduction in antinutrient content, increases amino acids, carbohydrates, fibers, polyphenol content, and antioxidant potential	NA	Germination	Gamel et al. (2006)
	Best way to maintain (and even improve) amaranth nutritional values	NA	Germinated flour at 30°C during 78 h of germination	Perales-Sánchez et al. (2014)
	Quick digestion of starch content and increase in glycemic index	NA	Grinding/roasting	Capriles et al. (2008)
Buck wheat	Increases acceptability score of biscuits	Addition of buck wheat flour	NA	Baljeet et al. (2010)
	Rich source of nutraceutical compounds	NA	NA	Li and Zhang (2001)
	Higher lysine, iron, copper, and magnesium content	NA	NA	Ikeda and Yamashita (1994)
	Antioxidant potential	NA	NA	Oomah and Mazza (1996)
	Reduced starch digestibility, lowering of glycemic index, anticholesterolemic properties of protein fraction, well-balanced amino acid composition, and good source of dietary fiber and minerals,	NA	NA	Pomeranz and Robbins (1972); Kayashita et al. (1997); Skrabanja and Kreft (1998); Tomotake et al. (2000); Skrabanja et al. (2001); Steadman et al. (2001a; 2001b); (Ikeda et al. (2006);
	Reducing high blood pressure, lowering cholesterol, controlling blood sugar, and preventing cancer risk	NA	NA	Fabjan et al. (2003)
	Improved capillary fragility, retarded development of diabetes, anti-lipoperoxidant activities, anti-cancer activity, anti-hyperglycemic effect, protective effects against hemoglobin oxidation, a mitigation effect on cardiovascular diseases, anti-oxidative property, anti-mutagenic activity, anti-inflammatory activity, mitigation of diabetes, suppression of protein glycation, anti-platelet formation property, anti-angiogenic effect, neuroprotective effect	NA	NA	Griffith et al. (1944); He et al. (1985); Odetti et al. (1990); Nègre-Salvayre et al. (1991); Deschner et al. (1991); Wang et al. (1992); Grinberg et al. (1994); Oomah and Mazza (1996); Aheme and O'Brien (1999); Guardia et al. (2001); Je et al. (2002); Nagasawa et al. (2003); Sheu et al. (2004); Guruvayoorappan and Kuttan (2007); Pu et al. (2007)
	Thiamin-binding proteins (TBP) isolated from buckwheat	Serve as B_1_ vitamin transporters in the plant and stabilize it during technological processing	NA	Mitsunaga et al. (1986)
	Improvement of true digestibility	NA	Hypothermal transformations	Christa and Soral-Śmietana (2008)
	Increased antioxidative potential	NA	Honey obtained from buckwheat flowers	Gheldof et al. (2003)
	Induced apoptosis in leukemia cells (0.5–100 μg/ml, *in vitro*), induced apoptosis in human solid tumor cells (6.25–50.00 μg/ml)	Buckwheat trypsin inhibitor	NA	Park and Ohba (2004); Wang et al. (2007)
	Coarse type of flour (mainly responsible for producing acceptable flavor) and a fine type of flour (responsible for binding particles to each other that are present in the buckwheat flour) are produced	Important for preparing buckwheat noodles with high palatability and acceptability rather than modern milling with a roll milling machine	Traditional stone milling	Ikeda and Ikeda (2016)
	Increased resistant starch contents	NA	Cooking	Kreft and Skrabanja (2002)
	Reduced glycemic index	Formation of amylase-resistant starch produced by heating	Cooking	Skrabanja et al. (2000)

**Table 6:** Anti-nutrients and processes to decrease anti-nutritional activity in minor-millets and pseudo-cereals.

Crop	Anti-nutrients	Processes to decrease effects	References
Finger millet	Phytate content	Seed germination decreases phytic acid High temperature short time (HTST) process reduces anti nutrients	Nirmala et al. (2000); Kumar et al. (2018)
	Tannins	Germination (leaching and soaking) reduces tannins Boiling and pressure cooking can reduce tannins	Nirmala et al. (2000); Platel et al. (2010); Platel and Srinivasan (2016); Kumar et al. (2018)
	Polyphenols and phytates	Dehulling followed by soaking	Pawar and Parlikar (1990)
	Phytates, phenols, tannins	Decortications, milling, soaking, malting, germination, fermentation, popping and cooking	Shibairo et al. (2014)
	Polyphenols	Thermal/hydrothermal treatments, germination, decortication and fermentation	Subba Rao and Muralikrishna (2002)
	Phytates, tannins	Germination, fermentation	Hemalatha et al. (2007a); Platel and Srinivasan (2016)
	Phytate	Treatment with fungal phytase	Hemalatha et al. (2007b)
	Tannin, phytic acid, oxalic acid	Popping	Mishra et al. (2014)
	Phytates, polyphenols, tannins	Soaking, germination, steaming, fermentation	Sripriya et al. (1997)
	Phytates, polyphenols, tannins Phytates	Malting	Platel et al. (2010)
		Decortication	Krishnan et al. (2012)
		Germination	Tatala et al. (2007)
	Phenolic acids	Sprouting, pressure cooking, open pan-boiling, microwave heating	Hithamani and Srinivasan (2014)
Foxtail millet	Phytate, tannin, polyphenols	Dehulling, soaking, cooking	Pawar and Machewad (2006)
	Phytate	Thermal processing, mechanical processing (decortication, milling and sieving), soaking, fermentation, germination, malting,	Hotz and Gibson (2007)
	Phytate	Germination, soaking, puffing, fermentation, enzymatic hydrolysis	Saleh et al. (2013)
	Polyphenols	Germination and steaming	Mounika et al. (2017)
	Phenolic compounds	Fermentation, malting and steeping, thermal processing	Kaur et al. (2019)
	Phytic acid	Roasting	Gahlawat and Sehgal (1994)
Quinoa	Saponins	Repeated washing or dehulling after harvest. Extrusion and roasting techniques. Wet technique: Strong washing in cold alkaline water. Dry technique: heat treatment, extrusion, roasting, mechanic abrasion Wet technique is recommended as dry technique by abrasive peeling can allow the loss of protein, vitamins and minerals	Koziol (1990); Mastebroek et al. (2000); Dini et al. (2001); Brady et al. (2007); Comai et al. (2007); Spehar (2007); Farro (2008); Paśko et al. (2009); Jancurová et al. (2009); Ruiz et al. (2017); Maradini et al. (2017)
	Phytate compound in grains	Soaking, germination, fermentation and cooking decrease phytate compound	Ruales and Nair, 1993a; Hurrell, 2004; Umeta et al., 2005; Repo-Carrasco-Valencia et al., 2010; Lazarte et al., 2015; Iglesias-Puig et al., 2015
	Phytic acid	Similar process as for reducing saponin as brushing and rinsing can reduce around 30%. Fermentation and cooking can degrade the activation of phytase	Ruales and Nair (1993b); Oliveira et al. (2003); Khattab and Arntfield (2009)
	Tannins	Cleaning and rinsing in water Adequate washing (to cook) can reduce the harmful effect	Chauhan et al. (1992); Jancurová et al. (2009); Borges et al. (2010)
	Trypsin inhibitor	Heat treatment, boiling, roasting, domestic techniques employed for food preparation can reduce trypsin inhibitor concentration	Ruales and Nair (1993b); Jancurová et al. (2009); Borges et al. (2010)
	Nitrates	Found in leaves mainly. If the consumption in amount is higher can be harmful, but nitrate content is lower	Lopes et al. (2009)
	Oxalates	Contained in leaves, stem roots and hypocotyl seeds Quinoa has lower levels of Oxalic acid	Lopes et al. (2009)
Amaranth	Phytate, Phenolic compounds, Trypsin inhibitors, Chymotrypsin inhibitors, Amylase inhibitor	Seeds cooked, popped/extrusion, germinated seeds at 30, 60 and 90°C	Gamel et al. (2006); Ferreira and Arêas (2010)
Buckwheat	Trypsin activity inhibitor, alpha-amylase activity inhibitor, Poly-phenol content, Phytic acid content	Steaming, baking, boiling treatment in seeds and seedlings sprouting after 24, 48, and 72 h	Kreft (1983); Ikeda et al. (1986); Ikeda et al. (1991); Ookubo (1992); Campbell (1997); Amarowicz et al. (2008); Zhang et al. (2015)
	Tannins	Germination and sprouting can reduce protease inhibitors and increase protein digestibility. Further, dehulling grains and roasting	Kreft (1983); Ikeda et al. (1986); Ikeda et al. (1991); Ookubo (1992); Campbell (1997); Handoyo et al. (2006)

Singh et al. (2018) showed that traditional knowledge practiced by farmers to roast finger millet decreases phytochemical composition, moisture, protein, and antioxidant action, but increases fat, ash, and fiber and improves the bioavailability of iron and calcium. The improvement of iron bioavailability to reduce anemia happens *via* the biochemical changes in fortification with ferrous fumarate, ferric pyrophosphate (6 mg/kg), and zinc oxide (50 mg/kg) in finger millet flour (Tripathi and Patel, 2010). The diversity of finger millet offers a rich source of several antioxidants and calcium in the grains as polyphenols (0.3‒3.0%) that possess hypoglycemic, hypercholesterolemic, and anti-ulcerative properties (Chethan and Malleshi, 2007). Growing research interest exists in finger millet that could be attributed to several bioactive compounds such as ferulic acid-rich arabinoxylans, ferulic acid, caffeic acid, quercetin, and flavonoids, which are bio-accessible and have multiple therapeutic effects (Udeh et al., 2017). Hithamani and Srinivasan (2017) demonstrated that the bioavailability of phenolic compounds extracted from finger millet grain and co-administered to rats with piperine had a therapeutic benefit. The wide spectrum of phenolic compounds greatly enhances the nutraceutical potential of finger millet. Unprocessed and processed finger millet flour use in wafer and vermicelli (a fine noodle) have shown that bio-accessibility of Fe, Zn, and Ca through *in vitro* digestibility of starch (IVSD) and protein (IVPD) and bioactive polyphenols and flavonoids could be changed just by processing (Oghbaei and Prakash, 2012).

#### Genomics of Finger Millet

Finger millet [*E. coracana* (L.) Gaertn.] is a self-pollinated allotetraploid (2n = 4x = 36, AABB) species with a genome size of 1.593 Gb (**Table 7**). The 2C DNA amount in *E. coracana* is 3.36‒3.87 picogram (pg) (Mysore and Baird, 1997). It is an annual C4 herbaceous cereal crop belonging to family Poaceae and sub-family Chloridiodeae and exhibits morphological similarity to *E. coracana* subsp. *africana* and *E. indica*. Cytological studies, isozyme chloroplast DNA, and genomic *in situ* hybridization (GISH) have shown that the maternal diploid genome (AA) of *E. coracana* originated from *E. indica* whereas *E*. *floccifolia* is supposed to be the donor of the B genome to the polyploid species *E*. *coracana* (Bisht and Mukai, 2001). Because of its resilient nature, it is widely grown in arid and semi-arid areas of India and Africa.

**Table 7:** Details of genome organization and other characteristics of underused crops.

Species	Ploidy level	Chr. no.	Approx. genome size	Genes annotated
*Eleusine coracana*	Allotetraploid	36	1.6 Gb	85,243
*Setaria italica*	Diploid	18	513 Mb	38,801
*Chenopodium quinoa*	Allotetraploid	36	1.5 Gb	62,512
*Amaranthus* sp.	Diploid	32, 34	466 Mb	23,059
*Fagopyrum esculentum*	Diploid	16	540 Mb	35,816
*Fagopyrum tataricum*	Diploid	16	540 Mb	33,366

##### Molecular Markers, Genetic Diversity, and Phylogenetic Studies in Finger Millet

Despite the nutritional benefits and climate-resilient nature of finger millet, the available genomic resources are limited, which has slowed the pace of genetic improvement of this crop (Saha et al., 2016). Immense morphological diversity is present in finger millet with a range of seed color correlated with protein and calcium content, time to maturity, and drought and salinity tolerance (Vadivoo et al., 1998; Tsehaye et al., 2006). Very few reports exist on the use of molecular markers for studying genetic diversity in finger millet, although the development and use of molecular markers in genomic studies of finger millet started a decade ago. Arya et al. (2009) developed 31 expressed sequence tag simple sequence repeats (EST-SSRs), out of which 17 were amplified and nine were found to be polymorphic between 11 elite germplasm accessions of finger millet of Indian and African origin. Reddy et al. (2012) identified 132 EST-based SSRs and developed 30 SSR primers for assessing genetic diversity in 15 finger millet accessions. Out of 30 EST-SSRs, 20 primers showed polymorphism and 13 primers were found to have polymorphism information content (PIC) value above 0.5. Using transcriptome data, Selvam et al. (2015) identified several SSRs and designed and validated 12 SSR primers on 23 finger millet accessions, where the primers showed an average PIC value of 0.67. Dida et al. (2007), using random *HindIII*, *PstI*, and *SalI* libraries, developed 82 genomic SSR markers and developed the first genetic map of finger millet using genomic SSRs, restriction fragment length polymorphism (RFLP), amplified fragment length polymorphism (AFLP), and EST markers. The map covered 721 centiMorgan (cM) on genome A and 787 cM on genome B and consisted of 18 linkage groups. Phylogenetic studies using 45 genomic SSRs on 79 finger millet accessions showed that finger millet was domesticated in Africa first and was then introduced to India (Dida et al., 2008). Apart from work by Dida et al. (2007), 49 new polymorphic genomic SSR markers were developed by Musia (2013) using next-generation sequencing (NGS) data. Gimode et al. (2016) sequenced two genotypes of finger millet (KNE755 and KNE796) using Roche 454 and Illumina technologies and identified 10,327 SSRs and 23,285 single nucleotide polymorphism (SNP) and tested 101 of each across a diverse set of wild and cultivated finger millet accessions. Several other research groups identified EST-SSRs using sequences deposited in the NCBI database (Arya et al., 2009; Reddy et al., 2012; Babu et al., 2014b). The mean PIC value for 49 polymorphic SSRs tested was 0.42, whereas the mean PIC value for 80 polymorphic SNPs was 0.29. Genetic diversity analysis using molecular markers has reported low polymorphism showing a narrow genetic pool of cultivated finger millet genotypes (Muza et al., 1995). Using 14 polymorphic genomic SSRs and three genic SSRs, Arya et al. (2013) showed that African accessions have higher genetic diversity than Indian finger millet accessions. Apart from SSR markers, Kumar et al. (2016) identified 23,000 SNPs using genotyping by sequencing of 113 finger millet genotypes.

##### Marker Trait Associations in Finger Millet

Finger millet has been reported to contain 5‒30 times higher calcium than other cereals (National Research Council, 1996) and 44.7% of the essential amino acids (Mbithi-Mwikya et al., 2000). Genetic diversity analysis of 103 finger millet genotypes using 36 EST-SSRs associated with *opaque2* modifiers and 20 SSR primers associated with calcium transporters and calmodulin genes differentiated the finger millet genotypes based on protein and calcium content (Nirgude et al., 2014). Cereal endosperm proteins lack essential amino acids such as lysine and tryptophan and *opaque 2* modifier (a bZIP transcription factor) is involved in regulating the accumulation of lysine and tryptophan in seed. A set of 67 functional SSR markers was developed and genetic diversity analysis in a global finger millet genotype collection for *opaque2* modifier genes classified the genotypes into three clusters with high, medium, and low tryptophan content with few exceptions (Babu et al., 2014c). Association mapping studies identified markers associated with various agronomic traits such as days to flowering, tiller number, plant height, blast resistance, finger number, etc. (Bharathi, 2011; Babu et al., 2014a; Babu et al., 2014b). Association mapping studies for nutritional quality traits identified two QTLs associated with tryptophan content and one QTL associated with protein content, and the marker associated with tryptophan content showed an inverse relationship with protein content in finger millet (Babu et al., 2014c). Further, nine markers were identified to be associated with calcium content (Kumar et al., 2015b). Apart from SSR and SNP markers, the orthologous genes for amino acid composition and calcium content in grains of finger millet were identified and the SSR variations within these genes among the accessions differing in protein and calcium content were used for developing gene-specific functional SSR markers (Reddy et al., 2011; Nirgude et al., 2014). Kumar et al. (2015a) carried out transcriptome analysis in developing spikes of finger millet and identified SSR motifs in the genes encoding calcium transporters and seed storage proteins. Despite the markers and genetic materials identified, progress in marker-assisted selection for genetic improvement of finger millet has lagged because of poor understanding about the complex traits to be transferred to the genotypes of interest.

##### Transcriptomes and Genomes of Finger Millet

A few transcriptomics studies have been carried out to have a better understanding about the complexity of traits in finger millet. Salinity-responsive transcriptome profiling using a next-generation sequencing platform (Ion Proton) in contrasting finger millet genotypes led to the identification of the genes/pathways involved in an improved salt tolerance mechanism (Rahman et al., 2014). To understand the underlying mechanism of calcium accumulation in grains, Kumar et al. (2015a) carried out transcriptome analysis in the developing spikes of two finger millet genotypes (GPHCPB 45, a high-calcium genotype, and GPHCPB 1, a low-calcium genotype) using Illumina Hiseq-2000. It has been hypothesized that the accumulation of calcium in different tissues and genotypes of finger millet varies due to the differential expression of the genes involved in uptake, translocation, and accumulation of calcium in different tissues. Mirza et al. (2014) carried out expression analysis of the genes involved in calcium translocation and storage in two contrasting finger millet genotypes for calcium content and observed a two-pore channel (TPC1) and Ca^(2+)^ ATPase that might be involved in calcium uptake and translocation, respectively, due to their strong expression in root, stem, and developing spikes; whereas Ca^(2+)^/H^(+)^ antiporter (CAX1) might be involved in calcium accumulation in seeds due to its over-expression in developing spikes. The correlation between expression of these genes and calcium accumulation shows that these genes can be further used for a biofortification program (**Table 8**).

**Table 8:** Details of important QTLs/genes linked with accumulation of nutritional/anti-nutritional factors in underused crops.

Crop	QTL/gene/enzymes	Trait	Reference
Finger millet	Two-pore channel (*TPC1*), Ca^2+^ ATPase, Ca^2+^/H^+^ antiporter (*CAX1*)	Calcium content	Mirza et al. (2014); Kumar et al. (2015a)
	27-kDa c-zein gene of opaque 2 modifier	Tryptophan content	Babu et al. (2014b)
	bZIP transcription factor RISBZ1	Protein content	Babu et al. (2014b)
Foxtail millet	Granule-bound starch synthase 1	Amylose content	Fukunaga et al. (2002); Bai et al. (2013)
Quinoa	Diaminopimelate aminotransferase, diaminopimelate epimerase	Lysine content	Zou et al. (2017)
	Pyridoxal 5′-phosphate synthase, dihydrofolate synthase, tetrahydrofolate synthase	Vitamin B_6_ and vitamin E content	Zou et al. (2017)
	Triterpene saponin biosynthesis activating regulator (*TSAR1* and *TSAR2*)	Saponin content	Jarvis et al. (2017)
	Granule-bound starch synthase I	Amylose content	Brown et al. (2015)
Amaranth	Cytochrome P450, 4,5-DOPA dioxygenase extradiol 1	Betalain content	Lightfoot et al. (2017)
	Aspartate kinase 1 and dihydrodipicolinate synthase	Lysine content	Clouse et al. (2016)
Buckwheat	Granule-bound starch synthase 1	Amylose content	Yasui et al. (2016)

To unravel and understand the complex genome of finger millet, two independent research programs were started for carrying out whole-genome sequencing of finger millet. These attempts were carried out by the Indo-Swiss collaborative program funded by the Department of Biotechnology, Ministry of Science and Technology, India, and coordinated by the University of Agricultural Sciences, Bangalore, India, in partnership with Functional Genomics Center Zurich (FGCZ), University of Zurich. The researchers sequenced the genome and transcriptome of a drought-tolerant and blast-resistant finger millet genotype (ML-365) using Illumina and SOLiD sequencing technologies (Hittalmani et al., 2017). The sequenced genome consisted of 1,196 Mb covering ~82% of the total genome size. Genome analysis revealed the presence of 85,243 genes and 49.92% of the genome was found to be consisting of repetitive DNA. Hatakeyama et al. (2018) carried out whole-genome sequencing and assembly of finger millet (cv. PR-202) using Illumina NextSeq500 and PacBio RS II systems. The assembled genome was found to be of 1,189 Mb, estimated to be covering 78.2% of the finger millet genome. Genome analysis resulted in the identification of 62,348 genes, of which 91% were functionally annotated. The whole-genome information on ML-365 and PR-202 can be used for candidate genes and marker identification, which can be further used in marker-assisted breeding programs for the genetic improvement of finger millet.

### Foxtail Millet

Foxtail millet (*S. italica* (L.) P. Beauv.), also called Italian, German, Hungarian, or Siberian millet, is a nutritional and natural staple used in many countries of East Asia for a long time. It has been cultivated in India, China, and many countries in Southeast Asia and Africa for many millennia and is quite popular in arid zones (Austin, 2006; Cheng and Dong, 2010; Mal et al., 2010). The grain of foxtail millet is quite rich in protein, fiber, and phosphorus compared with that of other minor millets (Muthamilarasan and Prasad, 2015). These grains are very rich in vitamin B_1_, B_3_, and B_5_ (**Table 4**), and also contain a much higher amount than other minor millets and common cereals such as rice, wheat, and maize (Cheng and Dong, 2010). Foxtail millet also contains all the EAA but has isoleucine, leucine, methionine, phenylalanine, threonine, tryptophan, and valine in significantly higher amounts (**Table 3**). This makes foxtail millet a highly important nutritive crop with rich genetic diversity for glutinous and non-glutinous grains with different lipid composition (Taira and Miyahara, 1983). Significant phenotypic variations provide ample opportunity for allele mining and the use of molecular markers to supplement breeding programs. This crop is also one of the most important C4 panicoid crops known for its small genome size (~490 Mb), short life cycle, and self-pollinating nature, which make it an excellent model crop for evolutionary studies within the panicoid grass system (Lata and Shivhare, 2017).

Foxtail millet, vis-à-vis other gramineous crops, is a naturally drought-tolerant crop (Goron and Raizada, 2015). It is a quite resilient crop and is better adapted to marginal environments, especially arid regions. It is an extremely suitable food for type 2 diabetics due to its low glycemic index (GI) as its starch digestibility is much lower than that of wheat (**Table 1**). However, its nutrient bioavailability could be further increased if it were processed by boiling or steaming (Pawar and Machewad, 2006; Ren et al., 2016) (**Tables 5** and **6**). Genotypic differences exist for antioxidant activities and some specific varieties such as SiA-2593 were identified as therapeutic and functional foods (Shejawale et al., 2016).

Foxtail millet starch is of great interest among entrepreneurs owing to its flexibility to make gels using foxtail millet flour and divalent cations such as CaCl_2_ and FeSO_4_ (Nagaprabha and Bhattacharya, 2016). *S. italica* is considered one of the best minor millet crops for anemic and diabetic people, thus reinforcing nutrition security besides food security (Bandyopadhyay et al., 2017). Processed protein from *S. italica* has been reported as a potential source of food additive (Mohamed et al., 2009). Ample diversity is available in India, China, France, Japan, Kenya, and Mexico gene banks for foxtail millet (Dwivedi et al., 2012), which can be exploited for various nutritional traits, including protein, as the protein content is higher than that of other selected small millets (Rao et al., 2011). Besides this, its rich composition of beta-glucans (42.6%) present in the fiber helps to enhance sugar and cholesterol metabolism, which ultimately prevents diabetes and cardiovascular diseases (Kumari and Thayumanavan, 1997; Itagi et al., 2012; Muthamilarasan and Prasad, 2015). Efforts on genomics are in progress to identify the underlying factors for the mechanisms that improve nutritional factors in foxtail millet (Zhang et al., 2012; Muthamilarasan and Prasad, 2015). Traditional simple as well as advanced food processing helps to improve the bioavailability of micronutrients (iron, zinc, and proteins) and renders them in a form that is easy to assimilate by the body, along with a significant decrease in anti-nutrients (polyphenols and phytate) (Pawar and Machewad, 2006; Saleh et al., 2013). Anti-nutrients exist but grain processing reduces them drastically; however, omics can contribute a lot to improving the bioavailability of micronutrients and reducing anti-nutrients such as phytic acid, polyphenols, and tannins, to decrease processing cost and time.

#### Genomics of Foxtail Millet

Foxtail millet (*S. italica* (L.) P. Beauv.) is one of the oldest domesticated cereals in the Old World. The genus *Setaria* belongs to the subtribe Cenchrinae and tribe Paniceae within the subfamily Panicoideae (Kellogg, 2015). *Setaria* is the largest genus in the Cenchrinae, consisting of ≈100 species and all possessing the C4 photosynthetic pathway. The small diploid genome (2n = 2x = 18; 513 Mb), self-pollination behavior, and short life cycle (6 weeks) have made *Setaria* an ideal model plant for functional genomics studies in millets as well as in cereals (**Table 7**).

##### Molecular Markers, Genetic Diversity, and Phylogenetic Studies in Foxtail Millet

Among millets, *Setaria* is the most deeply studied genus at both the genetic and molecular level. Various types of molecular markers used for genetic diversity and phylogenetic studies in *S. italica*, including RFLP (Wang et al., 1998; Fukunaga et al., 2002), random amplified polymorphic DNA (RAPD) (Schontz and Rether, 1999), AFLP (d'Ennequin et al., 2000), and SSRs (Lin, 2012; Trivedi et al., 2018), showed that foxtail millet genotypes differed genetically between different regions and Chinese landraces were found to be the most variable compared with landraces from other places. Wang et al. (1998) first reported the RFLP-based map consisting of 160 loci using an intervarietal cross of foxtail millet (Longgu 25 × Pagoda Flower Green), which was later used by Devos et al. (1998) to construct a comparative genetic map of foxtail millet and rice. Seeing the importance of SSR markers, Jia et al. (2007) developed 26 EST-SSRs. Later, Jia et al. (2009), using two genomic libraries enriched for (GA)n and (CA)n, identified 100 polymorphic SSR markers and developed a linkage map using 81 SSRs and 20 RFLP-anchored markers. Later, Lin et al. (2011) developed 45 polymorphic SSR markers from a RAPD-enriched library and used them for genetic diversity analysis as well as proving their cross-species transferability. Gupta et al. (2011; 2012) developed 98 intron-length polymorphic (ILP) and 147 genomic SSR markers and further showed the high-level cross-species transferability. Considering the importance and ease of microsatellite markers in MAS because of their high reproducibility, co-dominant nature, multiallelic variation, and abundance in genome work, *Setaria* was used to mine the genome wide SSRs by analyzing the genome sequence information. The genome-wide analysis of foxtail millet resulted in the identification of 28,342 microsatellite repeat-motifs spanning 405.3 Mb of the genome. Among the identified microsatellites, primers for 21,294 were designed and 15,573 markers were mapped on nine chromosomes to develop a high-density physical map of foxtail millet (Pandey et al., 2013). The validation of 159 developed markers in eight accessions of *Setaria* sp. showed 67% polymorphism and 89.3% cross-genera transferability across millet and non-millet species. Muthamilarasan et al. (2013) developed 5,123 ILP markers and proved their applicability in germplasm characterization, phylogenetic studies, transferability across species, and comparative mapping in millets and bioenergy grass species.

##### Genomes and Transcriptomes of Setaria

A milestone in the area of *Setaria* genomics was the release of the reference genome of foxtail millet cultivar “Zhang gu” using whole-genome shotgun sequencing combined with the Illumina second-generation sequencer covering ≈86% of the genome (Zhang et al., 2012). The sequencing resulted in the generation of 16,903 contigs and 439 scaffolds covering a total length of 423 Mb. Further sequence analysis identified 38,801 annotated genes. Apart from “Zhang gu,” a photo-thermo-sensitive male sterile line (A2) was sequenced and comparison of both genomes resulted in the identification of many SNPs (542,322), small insertions/deletions (33,587), and structural variants (10,839) between the two cultivars. A linkage map was constructed using an F_2_ population derived from “Zhang gu” and “A2” using 759 markers consisting of 118 SNPs and 641 structural variants (Zhang et al., 2012). *S. italica* accession “Yugu1” and *Setaria viridis* accession “A10” were sequenced using the ABI3730xl capillary sequencer (Bennetzen et al., 2012). The assembled genome was found to be 396.7 Mb covering 80% of the genome and genome analysis identified 24,000‒29,000 expressed genes. To date, foxtail millet is the only millet whose genome assembly is available to a chromosomal scale.

Because of the abiotic stress-tolerant nature of foxtail millet, several functional genomics tools have been applied to dissect its stress-tolerant nature (Zhang et al., 2007; Jayaraman et al., 2008; Lata et al., 2010; Lata and Prasad, 2011;  Lata et al., 2011; Puranik et al., 2011; Puranik et al., 2013; Qi et al., 2013; Tang et al., 2017). Besides understanding the molecular basis of abiotic stress tolerance, GWAS studies have been carried out for mapping the QTLs underlying various agronomic traits in foxtail millet (Jia et al., 2013; Gupta et al., 2014; Jaiswal et al., 2019).

However, despite the enormous health benefits, few proper attempts have been made to understand the genetics and genomics of nutritional traits in foxtail millet. Resequencing of waxy landrace Shi-Li-Xiang (SLX) and fine mapping using an F_2_ population derived from SLX (waxy) × Yugu1 (non-waxy) identified a waxy locus harboring starch synthase-encoding *GBSS 1* gene. Sequence analysis of GBSS 1 showed transposable elements confirming its waxy nature (Bai et al., 2013). To dissect the genetics and genomics of nutritional traits, genome and transcriptome data can be used to select genes associated with various pathways involved in biosynthesis and regulation of storage compounds and these can be exploited for understanding their role in the accumulation of various nutritional compounds.

### Quinoa

Quinoa (*C. quinoa* Willd.), pronounced “keenwa,” is a dicotyledonous plant originated from South America, hence called an Andean grain. For seven millennia, Andean cultures from pre-Columbian time have been eating it and it has been part of their diet. It is actually a pseudo-cereal due to its morphological grain shape like grass crops. It is classified into five ecotypes, based on geographic adaptation in the center of diversity (Hinojosa et al., 2018):

- Valley = grown at 2,000 to 3,500 m.a.s.l. in Colombia, Ecuador, Peru, and Bolivia;
- Altiplano = grown at high altitudes of more than 3,500 m.a.s.l. around Titicaca Lake on the border of Bolivia and Peru;
- Salares = grown in the salt flats of Bolivia and Chile and having a high tolerance of salinity;
- Sea-level = grown in the low-altitude areas of southern and central Chile;
- Subtropical or yungas = grown in the low-altitude humid valleys of Bolivia and including late-flowering genotypes.

Quinoa is a highly resilient crop that not only has superior nutritional quality vis-à-vis common cereals but can also withstand environments where other crops have difficulty to grow (Choukr-Allah et al., 2016; Nanduri et al., 2019). Since 2013, when UN/FAO designated 2013 as the International Year of Quinoa, interest in this crop has increased markedly worldwide due to awareness, mainly because of its balanced nutritional profile and its potential as an alternative to feed the growing world population in a sustainable manner, especially when more food has to come from marginal environments (Jacobsen et al., 2013; Zurita-Silva et al., 2014). The natural selection process of quinoa cultivars took place under severe adverse conditions of the Andes, such as limited rainfall and extreme aridity (Martínez et al., 2009), and in salt-affected soils (Ruiz-Carrasco et al., 2011). That explains quinoa's built-in abiotic stress tolerance of aridity, salinity, highland, and frost; hence, it is well suited to marginal environments. Although quinoa is drought and salinity tolerant, it is sensitive to high-temperature stress. It can tolerate a wide range of temperatures (from −8°C to 35°C), but high temperature above 35°C during flowering results in a significant reduction in seed set and ultimately yield (Jacobsen et al., 2005). For example, studies in Italy (Pulvento et al., 2010), Morocco (Hirich et al., 2014), Germany (Präger et al., 2018), Portugal (Pires, 2017), India (Bhargava et al., 2006a), Egypt (Eisa et al., 2017), Mauritania (Bazile et al., 2016), and the United States (Peterson and Murphy, 2015; Walters et al., 2016) have reported that high temperatures reduce quinoa seed yield.

Quinoa has a unique balance between oil (4–9%), protein (averaging 16%, with high nutritional relevance due to the ideal balance of its essential amino acid content), and carbohydrate (64%) (Schlick and Bubenheim, 1996; Bhargava et al., 2006a; Vega-Gálvez et al., 2010) (**Table 1**). Lysine, one of the essential amino acids, which is usually much less in plant-based diets, is relatively high in quinoa, indeed very close to the standard set by FAO for human nutrition (**Table 3**). Because of its high starch content (51–61%), it can be used in the same way as cereals for flour production (Mastebroek et al., 2000; Ogungbenle, 2003; Repo-Carrasco et al., 2003; Bhargava et al., 2006a; Stikic et al., 2012). In addition, quinoa is a good source of vitamins, oil (high in omega 3, linoleic and linolenic acids, 55–66% of the lipid fraction), and natural antioxidants such as α and γ tocopherol, and it has more minerals such as Ca, Fe, K, Mg, Cu, and Mn than other cereals (**Table 2**) (Repo-Carrasco et al., 2003; Vega-Gálvez et al., 2010; Fuentes and Bhargava, 2011; Stikic et al., 2012). The International Center for Biosaline Agriculture (ICBA), based in Dubai, has been working on the suitability of quinoa for marginal environments since 2006 and has found high Fe content vis-à-vis major cereals such as wheat, rice, and maize. Five improved quinoa genotypes from ICBA (Q1 to Q5) have been analyzed for Fe content and it ranged from 49.55 to 133 ppm depending upon growing conditions, showing high genotype-by-environment-by-management (G × E × M) interaction. Quinoa is highly suitable for eating by diabetics due to its low glycemic index (GI) (**Table 1**). Quinoa seeds release important compounds such as phytoecdysteroids and 20-hydroxyecdysone (20HE) while germinating. These released bioactive phytochemicals from the seeds can be an excellent staple for developing anti-diabetic food products as these bioactive phytochemicals can decrease the glucose level in the blood and are a potential source to treat obesity and hyperglycemia (Graf et al., 2014). Quinoa grains are also gluten-free and are considered as complete protein as they contain all nine essential amino acids that the human body cannot produce itself and they have very high lysine content overall vis-à-vis other cereals (Jacobsen, 2003; Albugoch and Lilian, 2009; Alvarez-Jubete et al., 2010). Quinoa's exceptional nutritional qualities led NASA to include it as part of its astronauts' diet on long space missions. A NASA technical paper mentions that while no single food can supply all the essential life-sustaining nutrients, quinoa comes as close as any other in the plant or animal kingdom (Schlick and Bubenheim, 1996).

Despite these nutritional qualities, some antinutritional factors (triterpenoid glycoside) are present in quinoa. Saponins, when present in the seeds, confer bitterness. Natural occurrence of saponins in quinoa grain is usually higher but some native varieties have low saponin as well. Even though saponins can be removed by repeated washing or dehulling, this consumes additional resources on postharvest processing (**Table 6**). Enough genetic variation has been reported in saponin content in quinoa, varying from 0.2 g/kg in sweet genotypes to 11.3 g/kg in bitter genotypes based on dry matter (Mastebroek et al., 2000). Saponin is present in the seed coat and washed saponin solution from seeds could be used as a by-product in biopesticide and therapeutic compounds (Ruiz et al., 2017). Reducing saponin content could broaden quinoa production globally in a more economically sustainable manner. It is reportedly controlled by a recessive gene (*TSARL1*) and genotypes with low saponin could be developed using conventional breeding techniques with the help of MAS (Jarvis et al., 2017).

Additional mineral inhibitor can influence the bioavailability and bio-accessibility of minerals of quinoa. Plant-based diets usually have a low bioavailability of minerals (mainly zinc, iron, and calcium) due to the presence of inhibitors such as phytates and tannins that reduce absorption. Phytate (myo-inositol-6-phosphate) is the main inhibitor of zinc, iron, and calcium. Degradation of phytate is important to allow the bio-assimilation of minerals. Bioavailability of iron is reduced if the phytate/Fe molar ratio is higher than 1, and, for bioavailability of zinc, this can affect the relation of the phytate/Zn molar ratio when it is higher than 5 (Iglesias-Puig et al., 2015).

Quinoa is a rich source of minerals and it has much higher zinc (2.73–5.01 mg/100 g), iron (4.82–7.19 mg/100 g), and calcium (77.10–211.90 mg/100 g) than other cereals based on results from six varieties of quinoa from Chile (Miranda et al., 2012). Unfortunately, the levels of phytates were quite high in quinoa vis-à-vis other cereals. It is better if the phyate:zinc molar ratio (Phy: Zn) is <15 (Bindra et al., 1986), phytate:iron (Phy : Fe) ratio <1 (Hurrell, 2004), and phytate:calcium (Phy : Ca) ratio <0.17 (Umeta et al., 2005). Ratios above the desirable values indicate low bioavailability of the mineral in the grain. Therefore, phytate can affect the bio-assimilation of important minerals in food if the molar ratios are high above the critical values. However, germination, soaking, cooking, and fermentation decrease the phytate compound in grains of quinoa and allow the bio-assimilation of iron, although much washing to some extent reduces the vitamin and mineral content as well (Ruales and Nair, 1993a; Valencia et al., 1999; Lazarte et al., 2015) (**Tables 5** and **6**). Cooking does not affect the amount of soluble iron; rather, it increases 2–4 times after soaking and germination, 3‒5 times after fermentation, and 5‒8 times after fermentation of the germinated flour combined with reduced phytates (Valencia et al., 1999). Soluble iron thus could be available to anemic populations in processed products based on germinated quinoa and quinoa sprouts (Vega-Gálvez et al., 2010). Brady et al. (2007) compared quinoa flour when processed by steam pre-conditioning, extrusion, and roasting. Steam pre-conditioning had the least effect on the chemical profile of quinoa while extrusion and roasting changed the chemical profile a lot compared to raw quinoa. The extrusion and roasting techniques can reduce saponin and the bitter taste (**Table 6**). Bioactive polyphenols and flavonols in quinoa grains and sprouts can help to prevent oxidative stress (Paśko et al., 2009).

#### Genomics of Quinoa

*C. quinoa* is an annual pseudo-cereal. It is an allotetraploid with 2*n =* 4*x =* 36 chromosomes having an estimated haploid genome size (C-value) of 1.005‒1.596 pg (Bennett and Smith, 1991; Stevens et al., 2006; Bhargava et al., 2007a; Palomino et al., 2008; Kolano et al., 2012) (**Table 7**). Quinoa has mostly smaller metacentric chromosomes of 0.94‒1.60 µm (Bhargava et al., 2006b; Palomino et al., 2008). Several studies on molecular understanding and genomics of quinoa have been carried out considering its nutritional importance. Most of the genetics and genomics studies have focused on either understanding its genetic diversity or evolutionary history whereas limited attempts have been made for genetic improvement of quinoa through molecular approaches. Most of the breeding has been carried out through mass selection for selecting high‐yielding, early maturing quinoa varieties with low saponin content as well as tolerance of biotic and abiotic stresses.

##### Molecular Markers, Genetic Diversity, and Phylogenetic Studies in Quinoa

The genetic diversity and phylogenetic relationship studies have been carried out between cultivated species of quinoa and their wild relatives using various types of phenotypic, biochemical, and molecular markers. Wilson (1988a) used morphological and biochemical markers to study the genetic relationships between quinoa ecotypes and classified them into two broad groups: coastal types and Andean types (above 1,800 m.a.s.l.). The Andean ecotypes were further classified into northern and southern Andean quinoa. Further, phylogenetic study showed that the Altiplano was the center of origin of quinoa (Wilson, 1988b). Rojas et al. (2000) classified the 1,512 accessions of the Bolivian National Quinoa Collection into seven distinct groups using various morphological and agronomic traits. Bhargava et al. (2007b) studied genetic diversity in quinoa using morphological and quality traits and showed a high level of genetic variability among the accessions.

Random amplified polymorphic DNA (RAPD) markers were the first markers used to detect DNA polymorphisms among different quinoa accessions (Fairbanks et al., 1993; Ruas et al., 1999; del Castillo et al., 2007). Using RAPD markers, very low intraspecific variations were observed within *C. quinoa* (Ruas et al., 1999). del Castillo et al. (2007) studied the hierarchical structure of the genetic variation present in eight quinoa field populations from Bolivia and found that population structure was related to three major biogeographic zones: the northern and central Altiplano, the inter-Andean valley, and the southern Salar. Apart from RAPD, AFLP markers have been used to study genetic diversity in quinoa. Rodríguez and Isla (2009) used AFLP markers along with 20 phenotypic markers to characterize 14 accessions of quinoa and concluded that Chilean lowland germplasm might be genetically more diverse, and the germplasm clustered together into highland and lowland/coastal as earlier reported by Ruas et al. (1999). Using SSR-enriched library sequencing, 208 polymorphic SSR markers were identified for quinoa (Mason et al., 2005). Recently, because of their reproducibility and co-dominant nature, SSRs have been used to study genetic diversity in quinoa (Mason et al., 2005; Jarvis et al., 2008). Christensen et al. (2007) used 152 accessions of USDA and CIP-FAO quinoa collections for genetic diversity study using 35 SSR markers and found that the accessions from lowlands and highlands clustered together and identified the group of accessions that appears to be hybrid between lowland and highland. Later, the use of multiplex fluorescent SSR markers to understand the genetic diversity and phylogenetics of 59 quinoa accessions resulted in the separation of highland and lowland genotypes into two separate clusters (Fuentes et al., 2009). Recently, Zhang, T., et al (2017) carried out whole-genome resequencing of 11 quinoa accessions and identified various SSR, SNP, and Insertion/Deletion (InDel) markers. They further used the identified SSR and InDel markers to assess the genetic diversity of 129 quinoa accessions from the USDA collection. These studies using various types of markers showed that a strong population structure and huge genetic diversity exist among quinoa germplasm.

##### Linkage Maps of Quinoa

Seeing the importance of quinoa in food and nutritional security, several breeding programs for improving grain yield, earliness, disease resistance, and drought tolerance and reducing saponin content have begun. Molecular markers and linkage maps help in QTL mapping, which further helps in marker-assisted selection (MAS) for speeding up the breeding process. The first linkage map of quinoa was developed by Maughan et al. (2004) using 19 SSR, 6 RAPD, and 230 AFLP markers spanning 1,020 cM covering 60% of the genome, consisting of 35 linkage groups with an average marker density of 4.0 cM per marker. Later, Jarvis et al. (2008) developed 216 new SSR markers and a more enriched linkage map for quinoa by using new SSR and 75 AFLP markers consisting of 41 linkage groups covering 913 cM. Further, using two RIL populations, Maughan et al. (2012) mapped 511 SNPs across 29 linkage groups of quinoa spanning 1,404 cM with a marker density of 3.1 cM per marker. Recently, Jarvis et al. (2017) developed a high-density linkage map of quinoa consisting of 6,403 SNP markers through genotyping by sequencing (GBS) covering 2,034 cM on 18 linkage groups. Further, apart from molecular markers, several other genomic resources such as bacterial artificial chromosome (BAC) libraries and stress-responsive or tissue-specific transcriptome sequencing have been used, which has helped in gene discovery as well as identifying molecular markers. The first EST library of quinoa consisting of 424 ESTs was developed from seed and floral tissues (Coles et al., 2005). Stevens et al. (2006) developed a BAC library of quinoa using *BamHI* and *EcoRI* restriction enzymes consisting of 26,880 and 48,000 clones, respectively. Using the same BAC library, Balzotti et al. (2008) identified and characterized 11S globulin and 2S albumin seed storage proteins of quinoa, which they predicted were responsible for the relatively high protein content and ideal balance of amino acids in quinoa. Maughan et al. (2009) isolated and characterized the *Salt Overly Sensitive 1 (SOS1)* gene using a BAC library and reported that *SOS1* is constitutively expressed in quinoa, unlike in other cereals in which mainly it is either stress-inducible or shows tissue-specific expression. Later, Walsh et al. (2015) carried out phylogenetic analysis in quinoa based on the sequence information of two introns of *SOS1* and identified two distinct polyploid lineages.

##### Transcriptomes and Genomes of Quinoa

The advances in next-generation sequencing technology and computational bioinformatics have accelerated genomics and transcriptomics research in quinoa. In the past few years, transcriptome studies have been carried out in quinoa to understand the molecular basis of drought and salinity tolerance. A drought-responsive transcriptome analysis carried out in two genotypes of quinoa using the Illumina HiSeq 2000 platform led to the identification of the genes involved in imparting drought tolerance to quinoa (Raney, 2012). Morales et al. (2017) performed transcriptome analysis in drought-tolerant Chilean quinoa genotype R49 and identified the drought-induced genes and pathways involved in providing drought stress tolerance in quinoa.

Using next-generation sequencing platforms, three research groups have independently completed quinoa genome sequencing (Yasui et al., 2016; Jarvis et al., 2017; Zou et al., 2017). Using two next-generation sequencing platforms, Illumina HiSeq 2500 (for short high-quality reads) and PacBio RSII (for longer reads and gap filling), Yasui et al. (2016) sequenced and assembled the draft genome of a quinoa inbred (Kd). The draft genome was found to be of 1.1 Gb size consisting of 24,847 scaffolds. The annotated genome of “Kd” was found to consist of 62,512 protein-coding genes around 535.5 Mb, leaving 49.2% of the genome as repetitive sequences. Further, a freely accessible Quinoa Genome DataBase (QGDB; http://quinoa.kazusa.or.jp/) was developed.

Zou et al. (2017) developed another draft genome sequence of an inbred line of quinoa (Real) using Illumina HiSeq 2500 and PacBio RSII platforms. The estimated genome size was 1.49 Gb covering 90.2% of the nuclear genome. Annotation of the draft genome sequence resulted in the identification of 54,438 protein-coding genes, of which 95.3% were functionally annotated. Approximately 65.5% of Real's genome consists of repeat sequences, 85.6% of which were found to be transposable elements comprising mostly retrotransposons. Further, to investigate protein quality, researchers analyzed comparative lysine, phenylalanine, and isoleucine content in three protein families [albumin, globulin, and late embryo abundant (LEA) proteins] and found that the lysine content of quinoa was significantly higher in all three protein families than in the other cereals such as wheat, rice, or maize. The high lysine content was not only at the free amino acid level but also for amino acid usage in seed protein sequences due to the presence of a high copy number of genes encoding the enzymes involved in converting aspartate into lysine. They also found that the high vitamin B and vitamin E content in quinoa is due to the presence of a high copy number of gene-encoding enzymes (*pyridoxal 5′-phosphate synthase, dihydrofolate synthase, tetrahydrofolate synthase*) involved in vitamin B_6_ and dihydrofolate biosynthesis (**Table 8**). Further, Zou et al. (2017) reported that the genes involved in ion sequestration, ABA homeostasis, and signaling are responsible for enhancing abiotic stress tolerance in quinoa. Based on transcriptome analysis, Zou et al. (2017) proposed a model for salt accumulation in salt bladders. Since few genes were found to be differentially expressed in epidermal salt bladders, this suggests that most of the transporter genes are constitutively active in salt sequestration in the bladders and that regulation of ion transport in bladder cells in response to salinity occurs at the protein level through protein phosphorylation (Zou et al., 2017).

Jarvis et al. (2017) published a more complete genome sequence of coastal Chilean quinoa accession PI 614886 (QQ74) using PacBio RSII and Illumina HiSeq 2500 sequencing platforms. The genome was assembled into 3,486 scaffolds spanning 1.39 Gb consisting of 44,776 genes and approximately 64% repetitive sequences. Along with quinoa accession PI 614886 (QQ74), the authors resequenced 15 other quinoa accessions along with five accessions of *C. berlandieri* and two of *C. hircinum*, which are supposed to be immediate tetraploid ancestors of quinoa. They further produced 18 pseudo-chromosomes of quinoa consisting of 1.18 Gb (i.e. ~80% of the predicted ~1.45 Gb haploid genome size of quinoa). Jarvis et al. (2017) identified two genes encoding basic Helix‐Loop‐Helix (bHLH) transcription factors involved in regulating the triterpenoid biosynthetic pathway associated with production in quinoa. They identified the *triterpene saponin biosynthesis activating regulator like (TSARL1* and *TSARL2)* genes, of which *TASRL2* was expressed in roots but not in flowers or immature seeds, whereas *TSARL1* was over-expressed exclusively in seeds of bitter lines vis-à-vis sweet lines, suggesting that *TSARL1* might be the functional TSAR ortholog involved in regulating biosynthesis of saponin in bitter quinoa genotypes (**Table 8**). Further, sequence analysis showed that alternative splicing on *TSARL1* results in a premature stop codon, thereby translating a truncated protein with a compromised functional ability in forming homodimer to bind DNA for its activity.

### Amaranth

Amaranth (*Amaranthus* sp.) is an ancient crop whose domestication and cultivation date back to around 8,000 years ago in Mayan civilization of South and Central America. There is no concrete evidence for the origin of *Amaranthus*. It was used as a staple food along with corn (maize) and beans in Mexico starting 1,400 years ago; however, its production declined after the collapse of Central American culture (Alvarez-Jubete et al., 2010). *Amaranthus* sp. is a highly nutritive pseudo-cereal, rich in proteins, vitamins, and minerals. Because of its nutraceutical value and climate resilience, amaranth has been relaunched and is being promoted as a suitable crop for food and nutritional security (Lakshmi and Vimala, 2000). Amaranth's leaves are consumed as vegetables and its grains as cereal. The amaranth family is divided into two sections: Amaranthus saucer and Blitopsis dumort (Allen, 1961). Based on its use, it has been grouped into grain amaranth, vegetable amaranth, and ornamental and weedy amaranth (Sauer, 1967). Grain amaranth has four species*, A. hypochondriacus, A. cruentus, A caudatus,* and *A. edulis* (Martínez-Cruz et al., 2014), whereas vegetable amaranth belongs to the section Blitopsis and has two major species, *A. tricolor* and *A. lividis* (Pal and Khoshoo, 1972; Madhusoodanan and Pal, 1981). As evident from recent past publications, amaranth has been attracting the attention of food technologists for exploring its functional aspects after the United States National Academy of Sciences showed its high nutritional value and agronomic potential (Monteros et al., 1994; Ulbricht et al., 2009).

Amaranth is a climate-resilient, fast-growing cereal-like plant. Despite its potential nutritional value, it has been underexploited. Protein content in the grain of amaranth species ranges from 13.1% to 21.0% with an average of 15%, which is comparatively higher than that of other cereals (**Table 1**) (Mlakar et al., 2009). Its amino acid profile makes it an attractive protein source as it contains significantly higher lysine content (4.9‒6.1%), which is an essential amino acid limiting in most cereals (**Table 3**). Amaranth protein is also richer in sulfur-containing amino acids (≈4.4%), which are limiting in pulse crops (Bressani, 1989). It is considered that if it is consumed along with other cereals, it will provide a “balanced” source of protein (Saunders and Becker, 1984). The balanced amino acid content in amaranth grain protein is close to the optimum protein reference pattern in the human diet as recommended by FAO/WHO (O' Brien and Price, 2008). The balanced nature of amino acid composition in amaranth grain protein is due to the presence of ≈65% of the protein in the embryo and only ≈35% in the perisperm, whereas in other grains ≈85% of the protein is present in endosperm, which is poorer in essential amino acids (Senft, 1979; Betschart et al., 1981). Mainly three amino acids (leucine, isoleucine, and valine) are limiting amino acid in amaranth grain but are present in excess in other grains. Amaranth flour has high glycemic index, therefore it is suitable for blending with other cereals (**Table 1**). Amaranth and wheat flour in 25:75 ratio bring down the GI quite low with good nutritional balance. Similarly amaranth and maize flour in a ratio of 1:1 nearly reaches the perfect score of 100 on the nutritionist's scale and also the combination of amaranth in wheat flour improves the nutritional value of baked products (Saunders and Becker, 1984; Bressani, 1989). Apart from the balanced profile of amino acid content, amaranth protein has high digestibility (≈90%), which further improves the bioavailability of amino acids when ingested. Apart from having good amino acid composition, amaranth protein is gluten-free, making it a choice of food for patients with coeliac disease incidence.

Besides protein, amaranth grain has higher oil content (5‒10%) than other cereals (**Table 1**). Amaranth oil contains 76‒77% unsaturated fatty acids consisting of primarily linoleic acid (25‒62%), oleic acid (19‒35%), palmitic acid (12‒25%), stearic acid (2.0‒8.6%), and linolenic acid (0.3‒2.2%). The ratio of saturated to unsaturated fatty acid ranged from 0.26 to 0.31 in oil of amaranth grain. Amaranth lipid is unique due to the presence of high biologically active compounds such as squalene (2‒7%), tocopherols (≈2%), phospholipids (up to 10%), and phytosterols (up to 2%) (Becker, 1994; León-Camacho et al., 2001; Berghofer and Schoenlechner, 2002). Squalene is an unsaturated hydrocarbon and is known as an obligatory precursor of sterols and has been reported to have antibacterial properties and anti-oxidative and anti-tumor effects in carcinogenesis.

Amaranth grain contains ≈60‒65% of carbohydrate, of which starch constitutes ≈57% and total dietary fiber constitutes ≈8‒16% (**Table 1**). Amaranth starch mostly consists of amylopectins (≈89.0‒99.9%) and is therefore known as “waxy starch” with unique characteristics of high viscosity and high gelatinization temperature. Despite high amylopectin content, the starch granules of amaranth are smaller (0.8‒2.5 μm) than those of other grains (3‒34 μm), providing them with high water-binding capacity, higher swelling power, lower gelatinization temperature, and high resistance to amylases, making amaranth a preferential source of starch in the food industry (Williams and Brenner, 1995; Pal et al., 2002) as well as providing high solubility and digestibility. The dietary fiber content in pale-colored amaranth seeds was 8%, whereas it was 16% in black-colored amaranth grains (Mlakar et al., 2009). In the leaves of vegetable amaranth, fiber content ranged from 6.95% to 9.65%, with an average of 8.39% ± 0.1% (Shukla et al., 2006).

Amaranth grains are rich sources of the minerals iron (72‒174 mg/kg), calcium (1,300‒2,850 mg/kg), phosphorus (455 mg/kg), sodium (160‒480 mg/kg), magnesium (2,300‒3,360 mg/kg), and zinc (36.2‒40.0 mg/kg) as well as the vitamin riboflavin (0.19‒0.23 mg/100 g of flour), ascorbic acid (4.5 mg/100 g), niacin (1.17‒1.45 mg/100 g), and thiamine (0.07‒0.10 mg/100 g) (Becker et al., 1981; Rastogi and Shukla, 2013) (**Tables 2** and **4**). Apart from the seeds, the leaves of vegetable amaranth (*A. tricolor*) are rich in minerals and have an average content of potassium of 3.7 ± 0.26 g/kg, calcium 1.7 ± 0.04 g/kg, magnesium 2.90 ± 0.01 g/kg, zinc 791.7 ± 28.98 mg/kg, iron 1,233.8 ± 50.02 mg/kg, manganese 108.1 ± 3.82 mg/kg, and nickel 222.6 ± 9.51 mg/kg (Shukla et al., 2006). Amaranth has all the necessary daily required vitamins up to a significant level and is an excellent source for reducing vitamin deficiency (Graebner et al., 2004). The leaves of *A. tricolor* contain 0.83 ± 0.02 mg/kg vitamin A (carotenoids) and 112.3 ± 5.0 mg/kg ascorbic acid (vitamin C) (Shukla et al., 2006). Despite these nutritional benefits, amaranth grain contains some antinutritional factors that can limit its food application. Amaranth grain contains growth inhibitors such as phytic acid (0.3‒0.6%), saponins (0.09–0.10%), and tannins, etc.

In the recent past, amaranth has become a crop of interest for its high nutritive value and great potential as a functional food given its cholesterol-lowering effect observed in animal models (Plate and Arêas, 2002; Mendonça et al., 2009). Despite the 75% *in vitro* digestibility of amaranth protein, net protein use ranges from 33.5% to 46% (Aguilar et al., 2015). Hejazi et al. (2016) showed that, after germination treatment at 28°C for 48 h, the nutritional quality and digestibility of amaranth resulted in not only improved protein digestibility (84%) but also decreased phytic acid and oxalate. Gamel et al. (2006) and Ferreira and Arêas (2010) demonstrated that amaranth extrusion increased calcium bioavailability in rats and suggested that amaranth can be a complementary source of dietary calcium once its bioavailability is favorably modified by the extrusion process (**Tables 5** and **6**). Whittaker and Ologunde (1990) demonstrated that iron supplementation through amaranth increases hemoglobin content because of the more absorbable form of iron in amaranth. Subramanian and Gupta (2016) reported that administration of amaranth extract through an oral dose increases the level of NO^3^‒ and NO^2^‒ in plasma as well as in saliva. The bio-accessibility of phenolic compounds was accessed in five wild (*Amaranthus hybridus*, *Brachiaria brizantha*, *Panicum maximum*, *Rottboellia cochinchinensis*, and *Sorghum arundinaceum*) and two domesticated cereal (*Eleusine corocana* and a red variety of *Sorghum bicolor*) grains found in Zimbabwe and showed that *Amaranthus hybridus* had the highest intestinal bio-accessibility (95.4 ± 0.01%) vis-à-vis the other cereals tested (Chitindingu et al., 2015). Serna-Saldivar et al. (2015) recommended the use of *A. caudatus* grain flour in maize tortillas to improve the bioavailability of nutrients and to reduce diabetes as well as an anthelmintic. Apart from these, no published studies have directly compared the relative bioavailability of other nutritional components of amaranth.

#### Genomics of *Amaranthus* Species

Amaranth is a C4 pseudo‐cereal and can be cultivated in a wide range of environments since it has good tolerance of drought and salinity (Kong et al., 2009; Teng et al., 2016).

##### Molecular Markers, Genetic Diversity, and Phylogenetic Studies in Amaranth

The species of *Amaranthus* are difficult to characterize taxonomically because of the similarity existing among many of the species, small diagnostic parts, intermediate forms, and the broad geographic distribution (Mujica and Jacobsen, 2003). The taxonomic and phylogenetic position of the genus was investigated using various phenotypic and genotypic evaluations (Sauer, 1955; Stetter and Schmid, 2017). The most recent taxonomic classification divided it into three subgenera: *A. albersia, A. acnida*, and *A. amaranthus* (Mosyakin and Robertson, 1996; Costea and DeMason, 2001). Popa et al. (2010) used nuclear ribosomal DNA (rDNA) Internal Transcribed Spacer (ITS) regions for analyzing 92 accessions of *Amaranthus* and identified 12 out of 92 as weed. Xu and Sun (2001) reported low ITS divergence and poorly resolved phylogeny among the closely related taxa *A. cruentus, A. caudatus,* and *A. hypochondriacus,* and their putative wild progenitors *A. hybridus, A. quitensis,* and *A. powellii*. Chan and Sun (1997) used RAPD markers and reported that grain amaranths originated from a common ancestor, *A. hybridus.* Studies using isozyme markers have demonstrated a high degree of polymorphism between the populations, through which it may be possible to identify the intermediate stages of domestication.

Molecular markers are the essential tools in modern plant breeding research programs. The first attempt to develop and characterize the molecular markers for amaranth was carried out by Mallory et al. (2008), who sequenced 1,457 clones from microsatellite-enriched libraries and identified 353, out of which 179 were found to be polymorphic across the accessions of three grain amaranths. Maughan et al. (2009) applied a genomic reduction strategy with next-generation sequencing and identified 27,658 SNPs among four diverse amaranth accessions. *Amaranthus hypochondriacus* showed the maximum genetic diversity in terms of the number of polymorphic SNPs, whereas *A. cruentus* showed the lowest genetic diversity with 35 polymorphic SNPs only (Maughan et al., 2009). The reduced genetic diversity in *A. cruentus* is consistent with other studies carried out using different marker systems: SSRs, RFLPs, isozymes, and AFLPs (Chan and Sun, 1997; Xu and Sun, 2001; Mallory et al., 2008). Suresh et al. (2014) studied genetic diversity and population structure in 348 amaranth accessions belonging to 33 species using 11 SSR markers. The accessions were grouped into seven different clusters independent of species or geographic origin. The overall PIC value ranged from 0.436 to 0.898, with an average of 0.657, with observed heterozygosity from 0.056 to 0.876. The variation in 29 grain amaranth accessions was studied using 27 phenotypic and 16 RAPD markers, resulting in grouping of these accessions into five clusters at 87.5% similarity coefficient (Akin-Idowu et al., 2016). The molecular phylogeny of 94 accessions representing 35 *Amaranthus* species evaluated using SNP markers through genotyping by sequencing (GBS) showed that most accessions from the same species clustered together and they were classified into three subgenera with a few highly differentiated groups (Stetter and Schmid, 2017). It was also hypothesized that *A. hybridus* might be the ancestor of all three crop amaranth species and *A. quitensis* might be an intermediate between *A. hybridus* and *A. caudatus*.

##### Transcriptomes and Genomes of Amaranth

Despite the great potentiality of amaranth as a source of nutritional food, few efforts have been made to explore its genomics. Stress-responsive transcriptome analysis of *A. hypochondriacus* was reported to understand the mechanism and identify the genes involved in adapting the species to survive against environmental stresses (Délano-Frier et al., 2011). The first attempt to sequence the draft genome of *A. hypochondriacus used Illumina Genome Analyzer IIx with >100× coverage of estimated genome size of 466 Mb (* Sunil et al., 2014 *). The annotation of A. hypochondriacus* resulted in the identification of 24,829 protein-coding genes and 13.76% of the genome was found to consist of repeat elements. They further hypothesized that high lysine content in *Amaranthus* is due to the presence of only one ortholog of *aspartate kinase 1* gene and high expression of the *dihydrodipicolinate synthase* (*DHDPS*) gene in seeds. Recently, a high-quality whole-genome sequencing of agronomically important amaranth cultivar “Plainsman” (*A. hypochondriacus*) was carried out using the Illumina HiSeq platform (Clouse et al., 2016). The assembled genome consisted of 377 Mb in 3,518 scaffolds covering 80.9% of the estimated genome size. Further, the genome consisted of 48% of the repeat sequences, of which Copia-like retrotransposons were predominantly present. Annotation of the genome led to the identification of 23,059 protein-coding genes. Clouse et al. (2016) further resequenced seven accessions of grain amaranths (*A. hypochondriacus, A. caudatus,* and *A. cruentus*) along with *A. hybridus.* SNP-based phylogenetic analysis confirmed that *A. hybridus* is the progenitor species of grain amaranths. Further, the researchers generated a physical map spanning 340 Mb of the whole genome of *A. hypochondriacus.* In addition, Lightfoot et al. (2017) performed single-molecule, real-time sequencing using the PacBio RSII system to close assembly gaps and used chromatin interaction mapping (Hi-C) to scaffold contigs and thereby improved their own previously reported Illumina-based assembly to a chromosome-scale assembly. The 16 largest scaffolds contain 98.0% of the assembly and represent the haploid chromosome number (n = 16). These researchers further produced physical and genetic maps and identified the betalain locus consisting of *CYP76AD1* (*cytochrome P450*) and *DODA1* (*4,5-DOPA dioxygenase extradiol 1*) involved in betalain biosynthesis, which controls stem color (**Table 8**).

### Buckwheat

Buckwheat (*F. esculentum* Monch) is a versatile dicotyledon annual crop and has been grown for centuries for its grains as well as greens to be used as food, feed, vegetable, and fodder, although it was neglected during the 20th century in western countries because of the increased yield of wheat during the Green Revolution (Cawoy et al., 2008). Nonetheless, it is recognized as a potential functional food source in China, Japan, and Taiwan. It is considered as a pseudo-cereal because of its usage as a conventional cereal and its chemical composition (Campbell, 1997). There are several species of buckwheat, out of which only nine species have agricultural and nutritional importance, among which only two are used for food purposes: common buckwheat (*F. esculentum)* and tartary buckwheat (*F. tataricum)*. Common buckwheat is also known as “sweet buckwheat,” which is taller, has a thicker stem, and is a widely cultivated species in Asia, Europe, North America, and South Africa, whereas tartary buckwheat is mainly confined to the highlands of southwest China and the Himalayas (Ohnishi, 1993; Zhou et al., 2016).

In the recent past, buckwheat production has increased because of its nutraceutical properties and potential for use in the preparation of functional foods (Li and Zhang, 2001; Bonafaccia et al., 2003a; Bonafaccia et al., 2003b). Buckwheat is rich in nutrients and its seed contains 100‒125 mg/g of protein, 650‒750 mg/g of starch, 20‒25 mg/g of fat, and 20‒25 mg/g of mineral (Li and Zhang, 2001).

The protein content in buckwheat flour is higher than in commonly used cereals such as rice, wheat, millet, sorghum, maize, etc. Buckwheat protein primarily contains albumin (180 mg/g), globulin (430 mg/g), prolamin (8 mg/g), and glutelin (230 mg/g) (Javornik and Kreft, 1984; Ikeda et al., 1991; Ikeda and Asami, 2000). The amino acid profile of buckwheat protein is balanced compared with that of other cereals. Buckwheat protein is one of the richest in lysine (EAA) and arginine, which are generally limiting in other cereals (**Table 3**). The amino acid score for buckwheat protein is 100, which is highest among plant sources (Ikeda, 2002). Lys/Arg and Met/Gly ratios determine cholesterol-lowering effects and are lower in buckwheat than in most plants, suggesting that buckwheat protein should have cholesterol-lowering effects (Huff and Carroll, 1980; Sugiyama et al., 1985; Carroll and Kurowska, 1995). Consumption of food products derived from buckwheat could reduce the concentration of cholesterol in blood serum and glycemic and insulin indices (Skrabanja et al., 2001; Stokić et al., 2015). Buckwheat protein contains either no or very low gluten and is thus considered as gluten-free and is recommended to people suffering from coeliac disease. Despite these benefits, the major problem is the low digestibility of buckwheat protein in humans and animals due to the presence of anti-nutritional factors such as protease inhibitors (trypsin inhibitor) and tannins (Ikeda et al., 1986; Ikeda et al., 1991; Campbell, 1997). However, sprouting/germination of buckwheat seed reduces the protease inhibitors, thereby increasing protein digestibility (Kreft, 1983; Ookubo, 1992) (**Table 6**).

Starch is the major carbohydrate in buckwheat grain and it varies from 59% to 70% of the dry mass depending on climatic and cultivation conditions (Qian and Kuhn, 1999). Buckwheat seed starch contains 24% amylose and 76% amylopectin (Praznik et al., 1999) but it's digestibility is much slower (low GI) than other cereals like wheat, therefore good to be consumed by diabetic patients. Buckwheat flour is commonly used during fasting days in India.

The total dietary fiber in seeds varies from 5% to 11% (Izydorczyk et al., 2004). Buckwheat is the richest source of soluble carbohydrates such as fagopyrin and fagopyritols, which have been studied for their use in treating type II diabetes (Kawa et al., 2003). Buckwheat grains also contain from 1.5% to 4.0% lipids (Steadman et al., 2001a). Buckwheat is rich in minerals such as potassium, magnesium, and phosphorus (**Table 2**). Apart from this, buckwheat contains higher contents of zinc, copper, and manganese than other cereals (Ikeda et al., 1999; Steadman et al., 2001b). The bioavailability of zinc, copper, and potassium from buckwheat is high and 100 g of buckwheat flour can provide 13–89% of the recommended dietary allowance (RDA) for Zn, Cu, Mg, and Mn (Bonafaccia et al., 2003b). Buckwheat grains contain higher contents of vitamin B_1_ (thiamine), B_2_ (riboflavin), B_3_ (niacin and niacinamide), K (phylloquinones), and B_6_ and B_6_ (folates) than other cereals (**Table 4**). Tartary buckwheat contains higher vitamin B_1_, B_2_, and B_3_, whereas common buckwheat contains higher vitamin E (Ikeda et al., 2006). The contents of vitamin C, B_1_, and B_6_ can be further increased by germinating buckwheat. It has been reported that the content of vitamin C can be increased by up to 0.25 mg/g in buckwheat sprouts (Lintschinger et al., 1997; Kim et al., 2004). Buckwheat also contains trace elements such as selenium (0.0099–0.1208 mg/g), which provides resistance against cancer and AIDS (Shi et al., 2011). Besides being rich in high-quality protein and minerals, buckwheat is rich in many rare components such as flavones, flavonoids, phytosterols, fagopyrins, and thiamin-binding proteins, which are known to have healing effects against some chronic diseases. The presence of flavonoids such as rutin, quercetin, orientin, homoorientin, vitexin, and isovitexin in its leaves, flowers, seeds, and sprouts imparts buckwheat with nutraceutical properties (Zielińska et al., 2012; Raina and Gupta, 2015). Tartary buckwheat has higher flavonoid content (19.54 mg/g) than common buckwheat (0.28 mg/g) (Jiang et al., 2007). Buckwheat is the only grain crop with rutin content that is known to have antioxidant, anti-inflammatory, and anti-carcinogenic property and it reduces the fragility of blood vessels related to hemorrhagic diseases and hypertension in humans (Oomah and Mazza, 1996; Baumgertel et al., 2003). Whole buckwheat contains 2‒5 times more phenolic compounds, whereas buckwheat hull and bran contain 2‒7 times more antioxidant than oat and barley (Holasova et al., 2002; Zduńczyk et al., 2006). Because of its good nutritional profile and presence of various nutraceutical compounds with unique medicinal properties and adaptability toward harsh climatic conditions, buckwheat is a suitable alternative for food and nutritional security for marginal environments.

#### Buckwheat Genomics

Buckwheat (*Fagopyrum* sp.), with its origin in China, is a pseudo-cereal belonging to family Polygonaceae. The genus has 19 species, of which *F. esculentum* (sweet buckwheat) and *F. tataricum* (bitter buckwheat) are the two species in the buckwheat genepool being cultivated predominantly in most parts of the world (Krkošková and Mrazova, 2005). Most of the species in the genus are diploid (2n = 2x = 16), except for *F. cymosum* and *F. gracilipes* being tetraploid (2n = 4x = 32) (Chrungoo et al., 2012; Farooq et al., 2016), with an estimated genome size of 540 Mb (Nagano et al., 2000) (**Table 7**). Despite having so many species, the genetic diversity in buckwheat has become depleted during the past few decades due to changing cropping patterns and food habits. Approximately 5,000 accessions of buckwheat have been collected in Southeast Asia, representing about 52% of the world's collection. China has the largest collection of buckwheat (2800), followed by Russia (2116), Ukraine (1600), and India (1050) (Zhou and Zhang, 1995; IPGRI-APO, 1999; Zhang et al., 2004; Zhou et al., 2018). The number of germplasm collections might be overrepresented due to duplications because of the exchange of germplasm between organizations within and between countries. It is an underused crop but holds tremendous potential due to its short life cycle (≈60 days), ability to grow and survive at higher altitude, and high-quality protein content.

##### Molecular Markers, Genetic Diversity, and Phylogenetic Studies in Buckwheat

A significant amount of research has been carried out to understand the properties of buckwheat proteins, flavonoids, flavones, phytosterols, thiamin-binding proteins, and other rare compounds (Tomotake et al., 2002; Kreft et al., 2006; Zielinski et al., 2009). However, little effort has been made in the development of molecular markers and genomic resources for buckwheat. RAPD has been used to study the relationship and estimate the genetic diversity between different accessions of *Fagopyrum* species (Sharma and Jana, 2002a; Sharma and Jana, 2002b) as well as to identify molecular markers linked with the *homostylar (Ho)* gene associated with self-compatibility (Aii et al., 1998). Nagano et al. (2001) used AFLP markers to identify markers tightly linked to the homostylar region and designed two primers to develop locus-specific markers for further use in marker-assisted breeding. Using the AFLP marker system, five markers linked to shattering habit in buckwheat were identified and a linkage map around the *sht1* locus was constructed (Matsui et al., 2004). An interspecific linkage map was developed using *F. esculentum* and *F. homotropicum* covering 548.8 cM on eight linkage groups. Microsatellite markers showed comparatively higher polymorphism and expected heterozygosity than AFLP, RAPD, and RFLP markers (Powell et al., 1996). Konishi and Ohnishi (2006) developed 180 SSR markers by sequencing 2,785 clones, out of which 54 were found to be polymorphic. Ma et al. (2009) developed 136 new SSR markers for *F. esculentum* and used them for diversity analysis in related species of the genus *Fagopyrum*. However, out of the 136 SSRs, only 10 were found to be polymorphic on 41 accessions. Kishore et al. (2012) assessed genetic diversity and phylogenetic relationship in 75 accessions using 15 SSR markers and the estimated average gene diversity was 0.2098. Li et al. (2007) attempted to develop microsatellite markers for tartary buckwheat by constructing a genomic library enriched with (gT)n repeats. Apart from these markers, a couple of BAC libraries were constructed for *F. homotropicum* (Nagano et al., 2001) and *F. esculentum* (Yasui et al., 2008), which can be further used for identifying useful genes as well as developing markers. More efforts have been made to carry out research in molecular genetics and plant breeding in common buckwheat, but only fragmentary research efforts have been made in tartary buckwheat. Genetic maps have been developed for both *F. esculentum* (Yasui et al., 2004; Pan and Chen, 2010) and *F. tataricum* (Du et al., 2013) and QTLs governing photosensitivity (Hara et al., 2011) and stem length (Yabe et al., 2014) have been identified. However, these maps remain underused for mapping genes/QTLs due to the lack of enough informative markers distributed across all linkage groups. The whole genome *de novo* sequencing of “HeiFeng No. 1,” an elite tartary buckwheat cultivar, using Illumina HiSeq 2000 resulted in 204,340 contigs and 348 Mb of assembled sequences. Further, the sequence survey resulted in the identification of 24,505 SSR motifs. The researchers further carried out SSR fingerprinting of 64 accessions and predicted the population structure of tartary buckwheat (Hou et al., 2016).

##### Transcriptomes and Genomes of Buckwheat

Transcriptome analysis for floral structure (Logacheva et al., 2011), aluminum toxicity (Chen et al., 2017; Xu et al., 2017) and salt tolerance (Wu et al., 2017) has been carried out to understand the gene regulatory mechanism in buckwheat. Transcriptome analysis at 12 different developmental stages of seed development from fertilization to maturation led to the identification of 11,676 differentially expressed genes in tartary buckwheat (Huang et al., 2017). The candidate genes identified through various transcriptomic studies provide a rich genomic resource for further functional characterization for various traits in buckwheat through a reverse genetics approach.

The whole-genome sequencing of buckwheat (*F. esculentum* Moench) using Illumina HiSeq 2000 resulted in assembly of the genome in 387,594 scaffolds consisting of 1.17 Gb of data. Gene prediction analysis identified 35,816 annotated genes and developed the Buckwheat Genome DataBase (BGDB; http://buckwheat.kazusa.or.jp) (Yasui et al., 2016). Researchers further showed the applicability of genome sequence information in identifying genes involved in flavonoid, 2S albumin-type allergens biosynthesis, and *granule bound starch synthases (GBSSs*), and in controlling heteromorphic self-incompatibility. Zhang, L., et al (2017) sequenced the whole genome of tartary buckwheat (*F. tataricum* cv. Pinku1) using multiple Illumina NGS platforms and SMRT sequencing technology. A total 8,778 contigs assembled into 489.3 Mb (N50 = 550.7 kb), with maximum contig length of 6.64 Mb. The annotation of the genome resulted in the identification of 33,366 protein-coding genes. Further, the reference genome helped in identifying predicted genes involved in aluminum stress tolerance and abiotic stress responses as well as rutin biosynthesis and regulation. Rutin is a flavonoid with antioxidant property known for its ability to strengthen blood vessels, aiding in the usage of vitamin C and production of collagen. Identification of the genes involved in rutin biosynthesis will further help in increasing the rutin content in buckwheat by using modern genetic and genomics tools.

## Summary and Conclusions

Improving the climate resilience of crops is crucial to the future food and nutritional security of marginal areas. Climate change is no longer a mirage but a reality that is evident from rising global temperature and more frequent drought, hot, cold, and rain spells worldwide. Every day, a large chunk of normal areas is being transformed into marginal lands, thus significantly decreasing productivity and endangering food security. More than 2 billion people that depend on major staple crops such as wheat, maize, and rice are suffering from “hidden hunger,” which has resulted in either malnutrition, due to deficiency of minerals, vitamins, and essential amino acids, or obesity, due to the surfeit of energy-rich carbohydrates. All of these major cereals are unable to tolerate climatic aberrations and marginality because of significant abiotic stresses. The need of the hour is to identify the crops and their varieties that can offer robust resistance against the harsh conditions of marginal environments and sustain food and nutritional security. Indeed, the targets for both globally healthy diets and sustained food production have to be met for 10 billion people on this planet by 2050. Several underused “neglected crops” are considered as “minor crops” and have received less importance globally in terms of both production and research. The five crops (finger millet, quinoa, foxtail millet, buckwheat, and amaranth) reviewed in this manuscript are nutrient-dense and are rich sources of macro- and micro-elements (e.g. protein with balanced amino acids, essential amino acids, vitamins, minerals, etc.). Small amounts of the minor millets and pseudo-cereals in the daily diet of people can ensure “no malnutrition.” Apart from being nutritionally rich, these underused crops are climate-resilient and suitable for cultivation in marginal environments. These neglected crops have huge potential for food and nutritional security through sustainable agriculture in marginal areas. This will help farmers to maintain productivity against a backdrop of rising temperatures, higher salinity, and increasing water scarcity and provide a practical and sustainable way of adapting to climate change. Unfortunately, the depth of scientific research on the yield and quality improvement of these minor millets and pseudo-cereals is extremely inadequate vis-à-vis that on the major staples; hence, international funding is extremely important to support research programs on genetic improvement for yield and nutritional traits for these crops.

## Author Contributions

JR and ST contributed on bioavailability part while HR contributed for genomics part. RS conceptualized, organized and finally polished the manuscript. All authors listed have made a substantial, direct, and intellectual contribution to the work, and approved for publication.

## Funding

The work was supported by the International Center for Biosaline Agriculture (ICBA) Dubai, UAE.

## Conflict of Interest

The authors declare that the research was conducted in the absence of any commercial or financial relationships that could be construed as a potential conflict of interest.
